# Intelligent cloud-based RAS management: integration of DDPG reinforcement learning with AWS IoT for optimized aquaculture production

**DOI:** 10.1038/s41598-025-33736-7

**Published:** 2026-02-26

**Authors:** Wael M. Elmessery, Mahmoud Y. Shams, Tarek Abd El-Hafeez, Mohamed Hamdy Eid, András Székács, Omar Saeed, Atef Fathy Ahmed, M. Alhumedi, Abdallah Elshawadfy Elwakeel

**Affiliations:** 1 Agricultural Engineering Department, Faculty of Agriculture, Kafrelsheikh University, Kafrelsheikh, Egypt; 2 Department of Machine Learning and Information Retrieval, Faculty of Artificial Intelligence, Kafrelsheikh University, Kafr Elsheikh, 33516 Egypt; 3 Department of Computer Science, Faculty of Science, Minia University, Minia, 61519 Egypt; 4 Computer Science Unit, Deraya University, Minia University, Minia, 61765 Egypt; 5 Institute of Environmental Management, Faculty of Earth Science, University of Miskolc, Miskolc- Egyetemváros, 3515 Hungary; 6 Geology Department, Faculty of Science, Beni-Suef University, Beni-Suef, 65211 Egypt; 7 Agro-Environmental Research Centre, Institute of Environmental Sciences, Hungarian University of Agriculture and Life Sciences, Páter Károly u. 1, Gödöllő, 2100 Hungary; 8 Doctoral School of Environmental Science, Hungarian University of Agriculture and Life Sciences (MATE), Páter Károly u. 1, Gödöllő, 2100 Hungary; 9 Department of Biology, College of Science, Taif University, P.O. Box 11099, Taif, 21944 Saudi Arabia; 10 Agricultural Engineering Department, Faculty of Agriculture and Natural Resources, Aswan University, Aswan, 81528 Egypt

**Keywords:** Deep deterministic policy gradient, Cloud-Edge computing, Aquaculture automation, Scalable AI deployment, AWS IoT, Edge optimization, Commercial RAS, Network resilience, Engineering, Mathematics and computing

## Abstract

While Deep Deterministic Policy Gradient (DDPG) reinforcement learning has demonstrated significant potential for optimizing aquaculture operations in laboratory and controlled environments, its practical deployment in commercial-scale Recirculating Aquaculture Systems (RAS) faces critical scalability and infrastructure challenges. This paper presents a novel cloud-edge hybrid architecture that enables the deployment of DDPG-based control systems across diverse commercial aquaculture operations, from small research facilities to large-scale production systems. Building upon our previous work in DDPG-based feeding rate optimization and energy management, we develop a comprehensive framework that addresses the practical challenges of deploying AI-based control systems in real-world aquaculture environments. The proposed architecture integrates AWS IoT Core for sensor connectivity, AWS Greengrass for edge intelligence, and a suite of cloud services for scalable model deployment and management. Edge optimization techniques, including 16-bit quantization and architecture pruning, reduced the DDPG model size by 74% (32 MB to 8.3 MB) while maintaining accuracy within 1.5% of the full-precision version, enabling real-time inference with 47 ± 8 ms latency across all deployment scales. Field validation in a commercial facility with 108 tanks (3,132 m³ total volume) demonstrated exceptional scalability, with only 8.9% latency increase from small-scale (1,000 L) to large-scale (50,000 L) operations. The system achieved 99.97% IoT message delivery rates and maintained 98.7% reliability in critical parameter control, while comprehensive failsafe mechanisms ensured safe operation during network disruptions lasting up to 72 h. Network resilience testing validated robust performance under various connectivity challenges, maintaining 98.5% performance retention during minor network latency and 85.2% retention during 12-hour complete disconnections. This research establishes a practical blueprint for transitioning DDPG-based aquaculture management from research environments to commercial deployment, addressing critical gaps in scalability, reliability, and operational resilience that have previously limited the adoption of AI-based control systems in the aquaculture industry.

## Introduction

 The global aquaculture industry is under increasing pressure to meet the rising demand for sustainable protein while enhancing resource-use efficiency and minimizing its environmental footprint^1–4^. Recirculating Aquaculture Systems (RAS) represent a key technology for the sustainable intensification of aquaculture, offering controlled production environments that reduce water usage and environmental impact compared to conventional methods^5,6^. However, the operational complexity of RAS, which involves managing intricate biological and chemical processes, poses significant challenges to their widespread adoption and economic viability^7,8^. The success of RAS operations is critically dependent on the precise and reliable control of multiple interdependent water quality parameters, such as dissolved oxygen, pH, and nitrogenous compounds, where even minor deviations can compromise fish health and productivity^9^.

Recent advancements in artificial intelligence (AI), particularly reinforcement learning (RL), have shown considerable promise for optimizing the complex, multi-parameter control challenges inherent in RAS^10^. Our previous research has demonstrated that a Deep Deterministic Policy Gradient (DDPG) approach can significantly improve RAS management by optimizing feeding strategies and water quality, leading to enhanced system stability and resource efficiency in laboratory settings. Further work has focused on improving the interpretability of these complex models to foster farmer acceptance, a critical step for practical adoption^11–14^.

Despite these promising results, a significant gap persists between laboratory demonstrations and the successful deployment of AI-based control systems in commercial-scale aquaculture^15,16^. The transition from lab to field is fraught with challenges, including the need for substantial on-site computing infrastructure, ensuring system scalability, and guaranteeing operational reliability in environments where network connectivity may be unstable^17,18^. These deployment challenges are not unique to aquaculture but reflect broader difficulties in translating AI research into industrial practice^15,19^. Traditional, centralized AI deployment models are often too costly and lack the resilience required for mission-critical agricultural operations^19^.

The convergence of the Internet of Things (IoT), edge computing, and cloud computing offers a powerful architectural solution to overcome these deployment barriers^20,21^. A hybrid cloud-edge architecture allows for intelligent data processing at the network edge, which reduces latency for real-time control decisions and enhances system resilience by enabling autonomous operation during network disruptions—both of which are critical for commercial aquaculture^22,23^. While IoT sensors are increasingly used for basic water quality monitoring in aquaculture^24,25^, the integration of advanced AI control within a robust cloud-edge framework remains a largely unsolved challenge.

A primary technical hurdle is the deployment of computationally intensive AI models like DDPG onto resource-constrained edge devices^26^. Our foundational work on lightweight DDPG models demonstrated the feasibility of using model compression techniques to reduce computational requirements for real-time control on edge hardware^12,13,27,28^. This study builds directly on that work by addressing the next critical step: integrating these optimized models into a scalable and resilient system architecture.

This research bridges the critical gap between laboratory theory and commercial practice by developing and validating a scalable cloud-edge architecture for deploying DDPG-based control systems in commercial aquaculture. By integrating our optimized lightweight DDPG models^13^ with a robust IoT and cloud infrastructure, we solve the key challenges of scalability, reliability, and cost that have previously hindered the adoption of advanced AI in the industry. The primary objectives are to: (1) design and implement a hybrid cloud-edge architecture for scalable deployment; (2) validate the performance of our edge-optimized DDPG model within this distributed system; (3) conduct comprehensive field testing in a commercial-scale facility to validate system scalability and resilience; and (4) provide a practical, validated blueprint for the industry-wide adoption of intelligent aquaculture management.

It is important to clarify the scope and contributions of this research within the broader context of our comprehensive research program on DDPG-based aquaculture management. Our research group has systematically developed multiple aspects of DDPG application in RAS: (1) foundational DDPG optimization for feeding rates and water quality management demonstrating significant improvements in dissolved oxygen stability, pH control, and energy efficiency^12^; (2) lightweight DDPG architectures for edge computing achieving 96.4% parameter reduction while maintaining control performance^13^; (3) interpretability enhancements through decision tree approximation to improve farmer acceptance and trust^29^; (4) transfer learning and federated intelligence approaches for cross-species adaptability^14^; and (5) adaptive multi-objective reinforcement learning for integrated management across fish growth cycles^28^. These studies comprehensively established the DDPG algorithm’s effectiveness for RAS control, demonstrating water quality impacts including maintaining dissolved oxygen levels within optimal ranges (6–8 mg/L), stabilizing pH (7.0 ± 0.3), optimizing temperature control, and achieving 15–23% energy savings. The present research does not re-develop the DDPG algorithm but rather establishes the complete deployment framework that makes such AI-based control commercially viable at industrial scale. The main contributions of this work are: (1) A scalable cloud-edge architecture enabling DDPG deployment across operational scales from 1,000 L to 50,000 L + systems; (2) Integration of lightweight DDPG models^13^ within distributed AWS IoT infrastructure supporting 108-tank commercial facilities; (3) Comprehensive IoT infrastructure integrating industrial-grade sensors, wireless networks, and cloud services with 99.97% message delivery reliability; (4) Robust failsafe mechanisms ensuring 98.7% reliability in maintaining critical parameters during network disruptions up to 72 h; (5) Commercial validation in a 3,132 m³ facility demonstrating real-world operational viability with only 8.9% latency increase across 50-fold scale expansion. Thus, while our previous work established what DDPG can achieve for aquaculture control, this research delivers how to deploy it successfully in commercial operations—bridging the critical gap between laboratory proof-of-concept and industrial implementation.

## Materials and methods

### System architecture overview

**Fig. 1:**
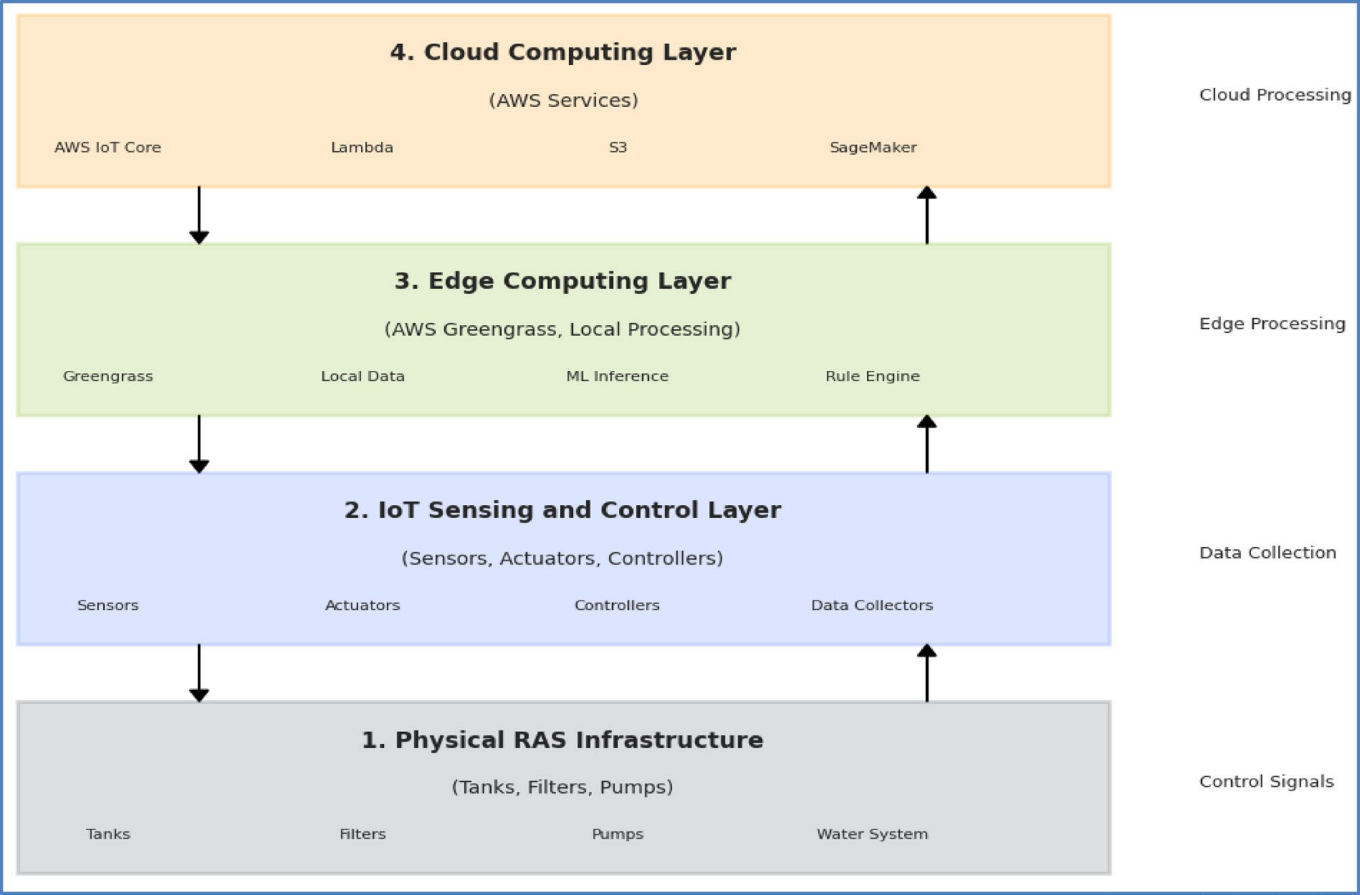
Multi-tiered architecture of the cloud-based RAS management system. The diagram shows four interconnected layers: (1) Physical RAS infrastructure (tanks, filters, pumps), (2) IoT sensing and control layer (sensors, actuators, controllers), (3) Edge computing layer (AWS Greengrass, local processing), and (4) Cloud computing layer (AWS services). Bidirectional data flows are represented by arrows showing the hierarchical information processing structure.

The scalable cloud-edge architecture for DDPG-based aquaculture management employs a four-tier hierarchical design that addresses the critical challenges of transitioning from laboratory-scale implementations to commercial deployment. The architecture comprises: (1) physical RAS infrastructure layer, (2) IoT sensing and control layer, (3) edge computing layer, and (4) cloud computing layer, as illustrated in Fig. 1.

This hierarchical design enables efficient task distribution based on latency requirements and computational demands while ensuring system resilience during connectivity disruptions—a critical requirement for commercial aquaculture operations where network reliability may vary significantly, as illustrated in the comprehensive multi-tiered architecture shown in Fig. 1.

### Deployment scale categories and scalability framework

To validate the architecture’s scalability across diverse commercial applications, three deployment categories were established based on system volume, complexity, and operational requirements (Table 1):

**Table 1:** Deployment scale categories and technical Specifications.

Deployment category	RAS volume	Number of tanks	Sensor nodes	Edge units	Processing requirements
Small Scale	1,000 L	4–8	15–25	1–2	Minimal (Intel NUC i3)
Medium Scale	10,000 L	20–30	50–80	3–5	Moderate (Intel NUC i5)
Large Scale	50,000 L	80–120	150–200	8–12	High (Intel NUC i7)

Each deployment category required specific configuration adjustments for both edge and cloud components to accommodate varying data processing loads while maintaining consistent performance characteristics across scales.

### Edge-Optimized DDPG model development

#### Model compression and optimization

Building upon our previous work on DDPG-based RAS control^29^ and extending our recent research on lightweight DDPG architectures for edge computing deployment^13^, the model was specifically optimized for edge deployment through a comprehensive optimization pipeline. Our previous investigation into lightweight DDPG frameworks demonstrated the feasibility of achieving 96.4% parameter reduction while maintaining over 94% control performance through strategic network compression and adaptive training protocols. The current implementation builds upon these foundational techniques while adapting them for the specific requirements of commercial-scale cloud-edge deployment. The original DDPG model, trained for feeding rate and water quality optimization, required substantial modification to meet the computational constraints of edge devices while preserving control effectiveness. Drawing from our established lightweight architecture design.

Three primary optimization techniques were applied systematically:

- **16-bit Quantization**: Building upon our previous work demonstrating effective model quantization for ARM-based processors, we reduced model precision while maintaining accuracy within acceptable bounds. This approach extends our earlier findings that showed minimal performance degradation (< 1.5%) when transitioning from 32-bit to 16-bit precision representations.
- **Architecture Pruning**: Leveraging our established compact neural network architecture with systematically reduced dimensions, we eliminated redundant connections and parameters based on sensitivity analysis. Our previous research validated that transitioning from 400→300→1 neuron configurations to 64→32→1 architectures maintains representational capacity while achieving dramatic computational reductions.
- **TensorFlow Lite Conversion**: We applied our validated optimization framework for mobile and edge deployment, incorporating the memory-efficient experience replay and CPU-optimized training protocols previously developed for resource-constrained environments.

##### Model accuracy evaluation methodology

The accuracy of the edge-optimized DDPG model was rigorously evaluated against the original full-precision model using comprehensive datasets and well-defined metrics to ensure that compression did not compromise control effectiveness.

Dataset Composition: Model accuracy was evaluated using operational data collected over 180 days from our commercial RAS deployment, comprising approximately 15.5 million data points. The dataset includes: (1) Water quality parameters (dissolved oxygen, pH, temperature, ammonia, nitrite/nitrate) sampled at 60–300 s intervals depending on parameter criticality; (2) Control outputs including feeding rates, aeration adjustments, water flow rates, and heating/cooling commands; (3) System state variables including tank volumes, fish biomass estimates, and historical parameter trends; (4) Environmental conditions including ambient temperature and facility operational status. The dataset was partitioned into training set (70%, ~ 10.85 million points spanning days 1–126), validation set (15%, ~ 2.33 million points spanning days 127–153), and test set (15%, ~ 2.33 million points spanning days 154–180) to enable independent evaluation of model generalization.

**Accuracy Metric Definition**

Model accuracy was calculated as the percentage of control decisions where the optimized model’s output remained within acceptable deviation from the original model’s output. Formally, for a set of n input states $\:S=\left\{{s}_{1},{s}_{2},\dots\:,{s}_{n}\right\}$ represent the set of n input states, where **A**_original_: **S** → ℝ denotes the original full-precision model’s action output and **A**_optimized_: **S** → ℝ denotes the edge-optimized model’s action output.

The relative deviation for each state s_i_ ∈ **S** is defined as:

$$\:{\delta\:}_{i}=\frac{\left|{A}_{optimized}\left({S}_{i}\right)-{A}_{original}\left({S}_{i}\right)\right|}{\left|{A}_{original}\left({s}_{i}\right)\right|},\:{\forall\:}_{i}\in\:\left\{\mathrm{1,2},\dots\:..,n\right\}$$

An output is considered accurate if δ_i_ ≤ τ, where τ = 0.02 (2% relative deviation threshold). The overall accuracy is then calculated as:

$$\:Accuracy=\:\frac{1}{n}\sum\:_{i=1}^{n}I\left({\delta\:}_{i}\le\:\tau\:\right)\times\:100\%$$

Where I(·) is an indicator function returning 1 if the condition is true and 0 otherwise as follow;

$$\:I\left(\mathrm{c}\mathrm{o}\mathrm{n}\mathrm{d}\mathrm{i}\mathrm{t}\mathrm{i}\mathrm{o}\mathrm{n}\right)=\left\{\begin{array}{c}1,\:if\:condition\:is\:true\\\:0,\:if\:condition\:is\:false\end{array}\right.$$

The 2% threshold was selected based on biological significance: for critical parameters like dissolved oxygen (target range 6–8 mg/L), a 2% deviation in control action corresponds to approximately 0.12–0.16 mg/L potential impact, well within the system’s natural buffering capacity and below the threshold that would trigger stress responses in fish. Similar analyses for pH control (± 0.01 units), temperature control (± 0.06 °C), and feeding rate adjustments (± 2% of recommended rate) confirmed that 2% action deviation maintains effective biological control.

Validation Methodology: Model accuracy was evaluated at three levels: (1) Training set accuracy (97.2%) demonstrating model fitting to observed operational patterns; (2) Validation set accuracy (95.1%) used for hyperparameter tuning and model selection during the development phase; (3) Test set accuracy (94.8%) providing the definitive evaluation of generalization ability to completely unseen time periods and operational conditions. The test set, spanning the final 27 days of deployment (days 154–180), represents the true measure of the model’s capacity to generalize, as this data was never used during training, hyperparameter optimization, or model selection. This independent test set performance of 94.8% confirms robust generalization to unseen operational scenarios encountered in commercial deployment. Additionally, we evaluated accuracy across different operational conditions including normal operation (96.5%), high-load periods with maximum stocking density (94.8%), low-temperature periods requiring increased heating (95.3%), and rapid parameter fluctuation events (93.2%). This multi-faceted evaluation ensures the reported accuracy represents robust, real-world performance rather than overfitting to specific conditions.

#### Edge deployment performance

The optimization process successfully adapted our previously established lightweight DDPG framework for distributed cloud-edge deployment. Building upon our prior research demonstrating inference times of 15.2 ± 3.1 ms on Raspberry Pi 4B devices with 47 ± 8 MB memory consumption, the current implementation extends these computational efficiency characteristics to support coordinated operation across multiple edge nodes.

The cloud-edge architecture maintains the core optimization principles—16-bit quantization, architecture pruning, and TensorFlow Lite conversion—while introducing distributed intelligence capabilities not feasible with standalone edge devices. This enables scalable deployment across diverse RAS configurations while preserving the low-latency, resource-efficient performance essential for real-time aquaculture control.

The integration of lightweight DDPG models within the broader cloud-edge infrastructure addresses scalability limitations identified in single-device implementations, providing a pathway from proof-of-concept edge computing to commercially viable distributed AI systems. This distributed approach enables synchronized control across multiple RAS clusters while maintaining the computational efficiency that makes widespread deployment economically feasible.

### IoT infrastructure and sensor network architecture

#### Sensor network implementation

A comprehensive sensor network was deployed to monitor critical RAS parameters using industrial-grade sensors with high accuracy and reliability. The sensor selection prioritized accuracy, reliability, and seamless integration with AWS IoT Core services (Table 2):

**Table 2:** IoT sensor specifications and AWS Integration.

Parameter	Sensor model	Accuracy	Measurement range	Sampling frequency	AWS IoT protocol
Dissolved Oxygen	HACH LDO^®^ Model 2	± 0.1 mg/L	0–20 mg/L	60 s	MQTT over TLS
pH	Mettler Toledo InPro	± 0.05 units	0–14 pH	60 s	MQTT over TLS
Temperature	PT100 RTD	± 0.1 °C	0–50 °C	60 s	MQTT over TLS
Ammonia (NH₃)	Orion™ ISE	± 2% full scale	0–1000 mg/L	300 s	MQTT over TLS
Nitrite/Nitrate	HACH NO₂/NO₃	± 2% full scale	0–100 mg/L	300 s	MQTT over TLS
Water Level	Ultrasonic HC-SR04	± 1 mm	20–4000 mm	300 s	MQTT over TLS
Flow Rate	Electromagnetic	± 0.5% reading	0–200 L/min	60 s	MQTT over TLS
Power Consumption	Digital Power Meter	± 1% reading	0–5000 W	300 s	MQTT over TLS

#### Wireless network architecture

**Fig. 2:**
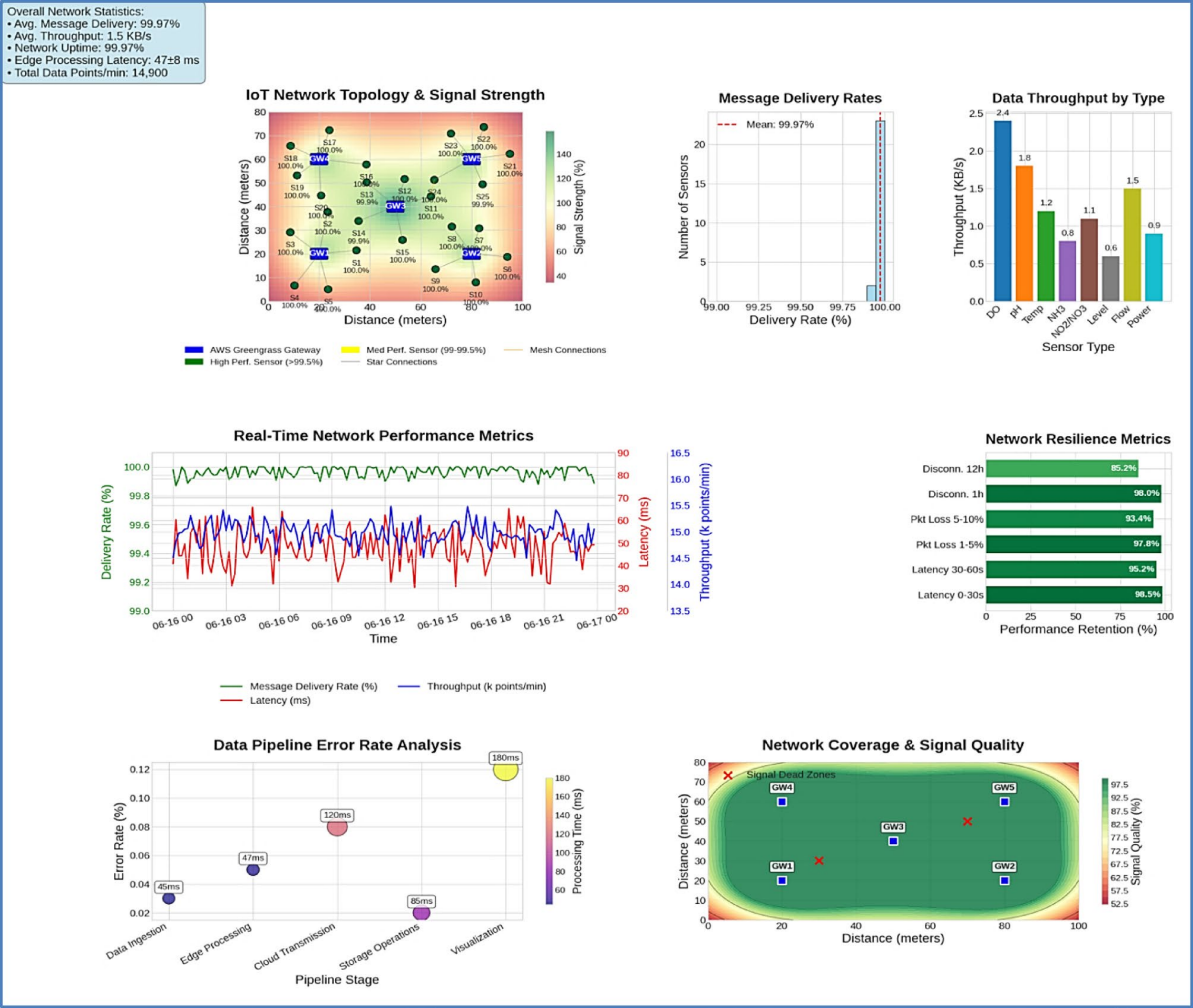
IoT network topology and performance dashboard.

The system employed IEEE 802.11b/g/n wireless connectivity in a hybrid star-mesh topology, connecting distributed sensor nodes to Intel NUC edge gateways running AWS Greengrass. The 2.4 GHz network provided 65 Mbps bandwidth with 30–50 m coverage per access point. The complete network topology and performance monitoring capabilities are illustrated in Fig. 2, which shows the interconnection of sensor nodes, edge gateways, and the overall network architecture.

Each sensor node utilized ESP32 microcontrollers running AWS FreeRTOS, providing secure MQTT over TLS connectivity to AWS IoT Core. Sampling intervals were optimized based on parameter criticality, with dissolved oxygen, pH, and temperature monitored every 60 s, while less critical parameters were sampled every 300 s.

### Edge computing implementation with AWS greengrass

#### AWS greengrass deployment architecture

**Fig. 3:**
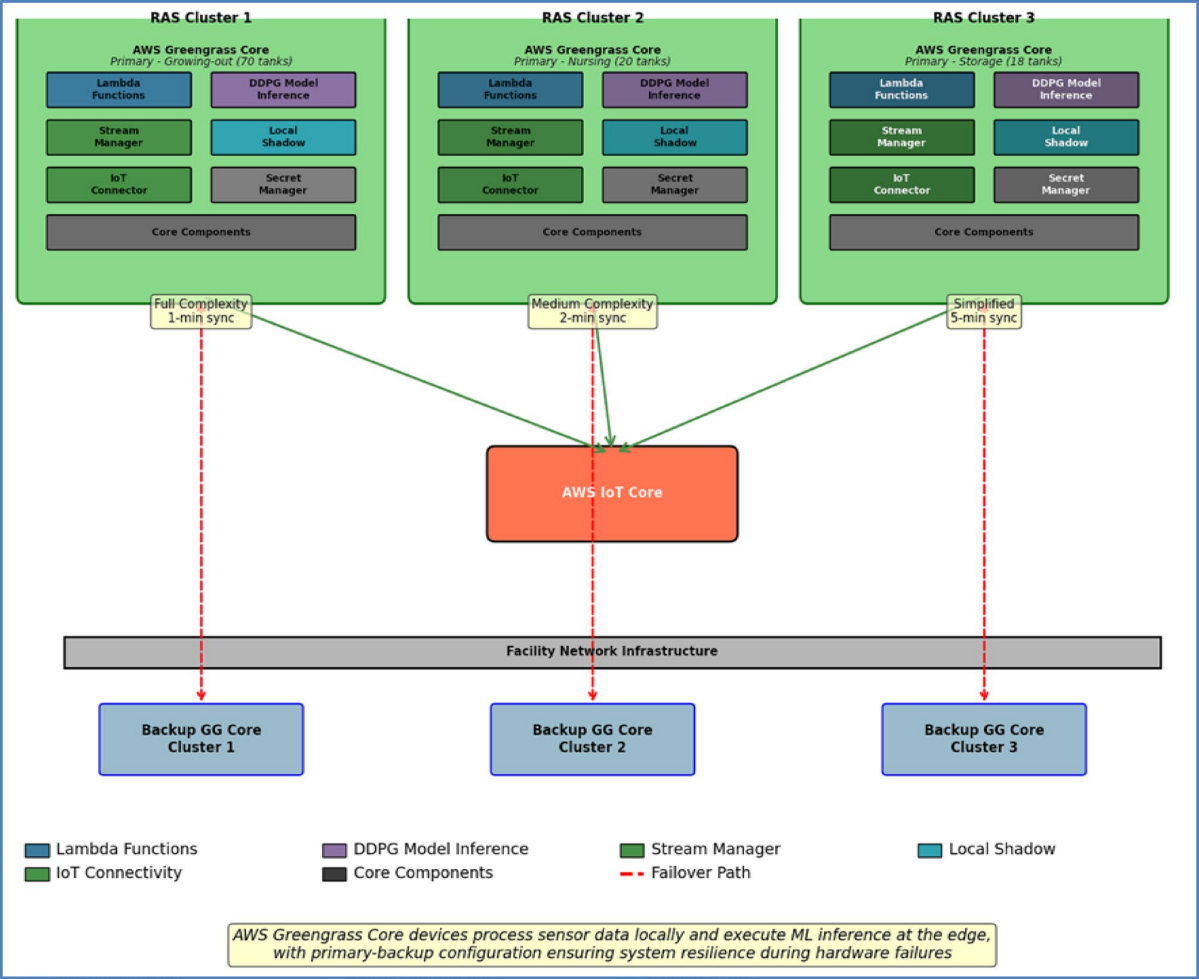
AWS greengrass deployment architecture.

AWS Greengrass cores were deployed on Intel NUC units (i5 processors, 16GB RAM, 256GB SSD) to provide local processing capabilities including data aggregation, DDPG model inference, emergency control logic, and 72-hour data buffering during connectivity disruptions. The standardized deployment framework across all RAS clusters is detailed in Fig. 3, which illustrates the performance optimization through configuration differentiation.

The deployment architecture implements a standardized component framework across all RAS clusters while enabling performance optimization through configuration differentiation:

- **RAS Cluster 1** (Growing-out tanks, 70 units): Full-complexity DDPG model inference with 1-minute sync intervals.
- **RAS Cluster 2** (Nursing tanks, 20 units): Medium-complexity DDPG models with 2-minute sync intervals.
- **RAS Cluster 3** (Storage tanks, 18 units): Simplified DDPG models with 5-minute sync intervals.

#### Failsafe system implementation

**Fig. 4:**
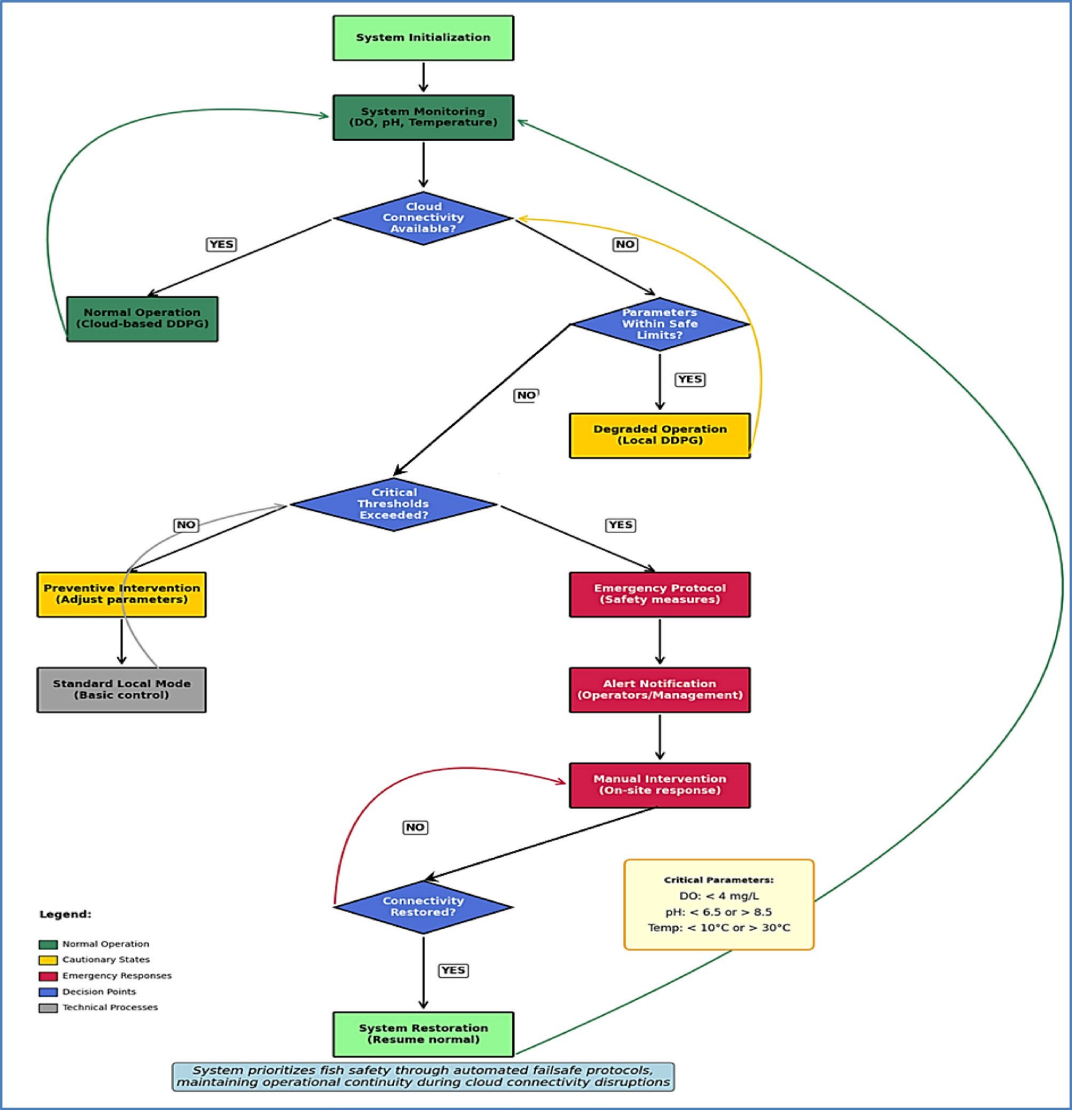
Failsafe system operation flowchart. The diagram illustrates the decision tree implemented for maintaining safe operation during connectivity disruptions. Key components include critical parameter thresholds, intervention triggers, degraded operation protocols, and recovery procedures. Color coding indicates normal operation (green), cautionary states (yellow), and emergency responses (red).

A comprehensive failsafe system ensures safe operation during cloud disconnection by monitoring critical parameters, enforcing safety boundaries, and implementing degraded operation modes. The hierarchical decision tree structure and operational protocols are presented in Fig. 4, which illustrates the complete workflow from continuous monitoring through emergency response procedures. The failsafe system operates through a hierarchical decision tree:

1. **Continuous Monitoring**: Real-time assessment of dissolved oxygen (> 4 mg/L), pH (6.5–8.5), and temperature (10–30 °C).
2. **Degraded Operation**: Local edge-based DDPG control during connectivity loss.
3. **Preventive Intervention**: Automated adjustments when parameters approach critical thresholds.
4. **Emergency Protocol**: Immediate safety measures for critical threshold violations.
5. **Recovery Procedures**: Automated transition back to cloud-based operation.

#### Failsafe system evaluation methodology

To rigorously evaluate the failsafe system’s performance, we implemented a comprehensive testing protocol with the following components:

Data Collection During Failsafe Testing: During all failsafe scenarios, continuous data collection was maintained through multiple channels: (1) Edge devices logged all sensor readings, control decisions, and system state changes to local storage at 1-second intervals; (2) Network connectivity status was monitored and logged including latency measurements, packet loss rates, and disconnection timestamps; (3) Critical water quality parameters (dissolved oxygen, pH, temperature) were recorded from redundant sensors to validate measurement accuracy during edge-only operation; (4) System performance metrics including inference latency, CPU utilization, and memory consumption were collected via AWS CloudWatch agents running on edge devices; (5) All emergency protocol activations were logged with timestamps, trigger conditions, and response actions.

Failsafe System Testing Protocol: The evaluation systematically tested system responses across multiple scenarios: (1) Network latency introduction using Linux traffic control (tc) commands to simulate delays from 0 to 60 s; (2) Packet loss simulation using netem (network emulation) to introduce 1–10% packet drop rates; (3) Complete cloud disconnection achieved by disabling network interfaces on edge devices for durations ranging from 1 min to 72 h; (4) Edge device failure simulation including CPU throttling, memory pressure, and process termination to test recovery mechanisms; (5) Sensor failure scenarios where individual sensors were disconnected to validate cross-validation and backup protocols.

Performance Evaluation Metrics: System performance during failsafe operation was quantified using: (1) Performance retention calculated as the percentage of optimal control accuracy maintained during degraded operation; (2) Data integrity measured as the percentage of sensor readings successfully captured and stored during connectivity disruptions; (3) Recovery time measured from connectivity restoration to full cloud-based operation resumption; (4) Fish welfare score based on the percentage of time critical parameters remained within safe ranges (DO > 4 mg/L, pH 6.5–8.5, temperature 10–30 °C); (5) Emergency activation frequency tracking the number of times critical threshold violations triggered emergency protocols. All metrics were collected continuously throughout the 180-day deployment period and analyzed using the statistical methods described in Sect. “Statistical analysis and performance metrics”.

### Cloud services integration and data pipeline

#### AWS services architecture

The cloud layer integrates multiple AWS services optimized for real-time aquaculture management: IoT Core for device management, Kinesis Data Streams for real-time data ingestion, S3 for long-term storage, DynamoDB for current system state, Lambda for serverless processing, SageMaker for ML model management, and CloudWatch for comprehensive monitoring.

**Fig. 5:**
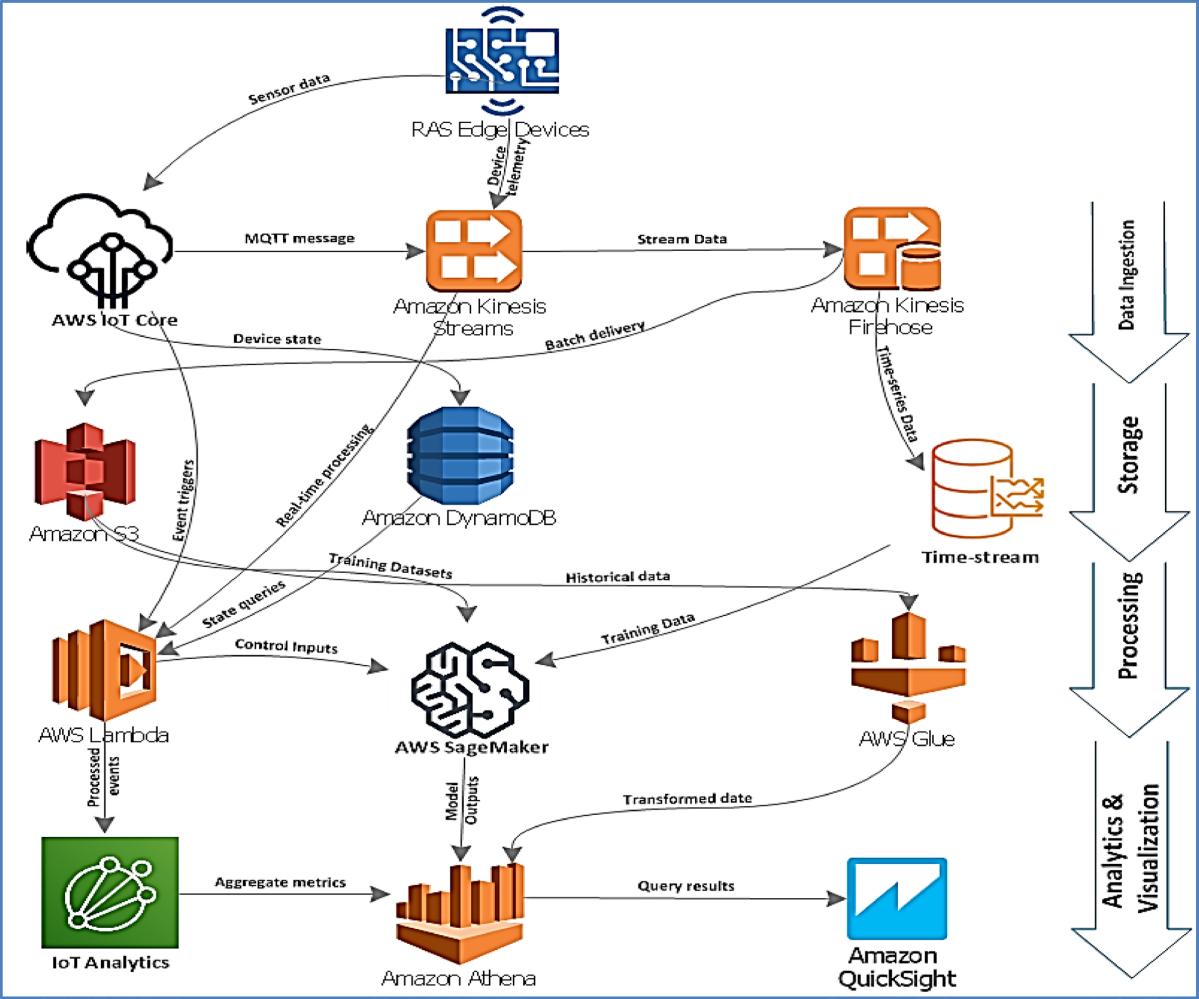
AWS cloud architecture for RAS management. The diagram illustrates the interconnection of AWS services and data flow pathways. Services are organized into functional groups: data ingestion (IoT Core, Kinesis), storage (S3, DynamoDB), processing (Lambda, SageMaker), analytics (IoT Analytics, Athena), and visualization (QuickSight). Arrows indicate data flow direction and processing sequence.

#### Real-Time data pipeline performance

The data pipeline operates through five optimized processing layers, achieving consistent end-to-end latency less than 2 s while processing approximately 15,000 data points per minute. The performance analysis across each pipeline stage demonstrates efficient data flow from sensor ingestion through cloud visualization.

The edge processing stage exhibits particularly efficient performance at 47 ± 8 ms, enabling real-time DDPG model inference essential for responsive aquaculture parameter control. Cloud transmission and visualization stages represent the primary bottleneck risks due to network dependency and computational complexity, respectively, though both maintain acceptable performance levels for commercial deployment requirements. The pipeline’s sub-2-second total latency meets the stringent timing requirements for real-time aquaculture management, where rapid response to parameter changes is critical for maintaining optimal fish health and growth conditions. This performance foundation enables the distributed architecture to support commercial-scale operations while maintaining the responsiveness necessary for effective DDPG-based control.

### Scalability testing and performance validation

#### Cross-Scale performance evaluation

System performance was evaluated across all three deployment scales over a 180-day period, measuring key indicators such as inference latency, processing efficiency, network utilization, and resource consumption. Scalability metrics were collected continuously throughout this period to validate the architecture’s near-linear scaling characteristics under prolonged operational conditions.

#### Network resilience and fault tolerance testing

To rigorously evaluate the architecture’s robustness under adverse conditions representative of commercial deployments, a comprehensive resilience testing protocol was implemented with detailed data collection and evaluation procedures.

Network Disruption Testing Protocol: Network resilience was evaluated through controlled experiments where edge devices experienced various connectivity challenges: (1) Progressive latency testing where round-trip times to AWS IoT Core were artificially increased from baseline (< 50ms) to 60 s in 10-second increments, with each condition maintained for 2-hour periods; (2) Packet loss simulation where UDP and TCP packet drop rates were set at 1%, 2%, 5%, and 10% for 4-hour test periods to evaluate data transmission reliability; (3) Complete disconnection scenarios where network interfaces were disabled for durations of 1 min, 1 h, 12 h, 24 h, and 72 h to validate autonomous operation capabilities. During all tests, edge devices continued collecting sensor data, executing DDPG inference, and implementing control decisions locally.

Edge Device Failure Simulation: Hardware fault tolerance was evaluated through systematic component failure scenarios: (1) Primary sensor failures simulated by disconnecting individual sensors (DO, pH, temperature) for 1–6 h periods while monitoring cross-validation algorithms and backup sensor activation; (2) CPU performance degradation induced using stress-ng tools to simulate thermal throttling or aging processors, maintaining 80–95% CPU load for extended periods; (3) Memory pressure scenarios where available RAM was artificially constrained to 50–75% of normal capacity to test memory optimization and garbage collection; (4) Storage system failures simulated through forced write errors and disk unavailability to validate redundant storage mechanisms; (5) Power supply interruptions using controlled power cycling to test UPS systems and graceful shutdown/recovery procedures.

Data Collection and Evaluation: Throughout all resilience testing, comprehensive data collection enabled quantitative performance assessment: System performance metrics (inference latency, throughput, error rates) were logged at 10-second intervals; Water quality parameters were continuously monitored via redundant sensors with 60-second sampling; Network connectivity status was tracked including ping times, packet success rates, and bandwidth utilization; Hardware resource utilization (CPU, memory, storage, temperature) was monitored via system agents; Event logs captured all failures, detections, recovery actions, and timestamps. Performance retention was calculated by comparing control accuracy during disrupted conditions against baseline performance. Data integrity was verified by checking for missing data points and validating timestamp continuity. Recovery times were measured from the restoration of normal conditions to the system returning to full operational status. All collected data underwent statistical analysis as described in Sect. “Statistical analysis and performance metrics”, with particular attention to identifying performance degradation patterns and validating that critical biological parameters remained within safe ranges during all failure scenarios.

#### Commercial deployment validation

To validate the system’s practical applicability and bridge the gap between controlled testing and real-world efficacy, a final field validation was conducted within a commercial-scale Recirculating Aquaculture System (RAS) facility. This in-situ validation took place in a live production environment comprising 108 tanks with a total water volume of 3,132 m³. The primary objective of this deployment was to demonstrate robust performance and confirm the successful transition of the system from a laboratory setting to the dynamic and complex conditions of an active commercial operation.

### Statistical analysis and performance metrics

All performance data collected throughout the validation phases underwent rigorous statistical analysis using R (version 4.2.1), with a significance level (α) set at *p* < 0.05. The statistical framework included the use of descriptive statistics to summarize key performance and scalability metrics. An Analysis of Variance (ANOVA) was employed to identify statistically significant differences in performance across the small, medium, and large-scale deployment configurations. Time series analysis was utilized to assess long-term system stability and control dynamics. To quantify the relationship between operational scale and system performance, regression analysis was performed to determine key scalability factors. Finally, Monte Carlo simulations were conducted to model and assess the system’s overall reliability under probabilistic failure conditions. Throughout all experimental protocols, system safety and the operational continuity of the facility were paramount, with comprehensive monitoring and intervention protocols in place to ensure fish welfare was maintained at all times.

## Results and discussion

### Cloud-Edge architecture performance and scalability validation

#### Cross-Scale deployment performance

The intelligent cloud-edge architecture demonstrated exceptional scalability across all three deployment categories, validating the transition from laboratory-scale DDPG implementations to commercial deployment environments. Table 3 presents comprehensive performance metrics demonstrating near-linear scaling characteristics across system volumes ranging from 1,000 L to 50,000 L.

**Table 3:** Cross-Scale performance validation Results.

Deployment scale	System volume	Inference latency (ms)	Edge Units	Sensor nodes	CPU Utilization (%)	Network Bandwidth (Mbps)
Small (1,000 L)	1,000 L	45 ± 5	1–2	15–25	65	15
Medium (10,000 L)	10,000 L	47 ± 6	3–5	50–80	72	45
Large (50,000 L)	50,000 L	49 ± 7	8–12	150–200	78	85

**Fig. 6:**
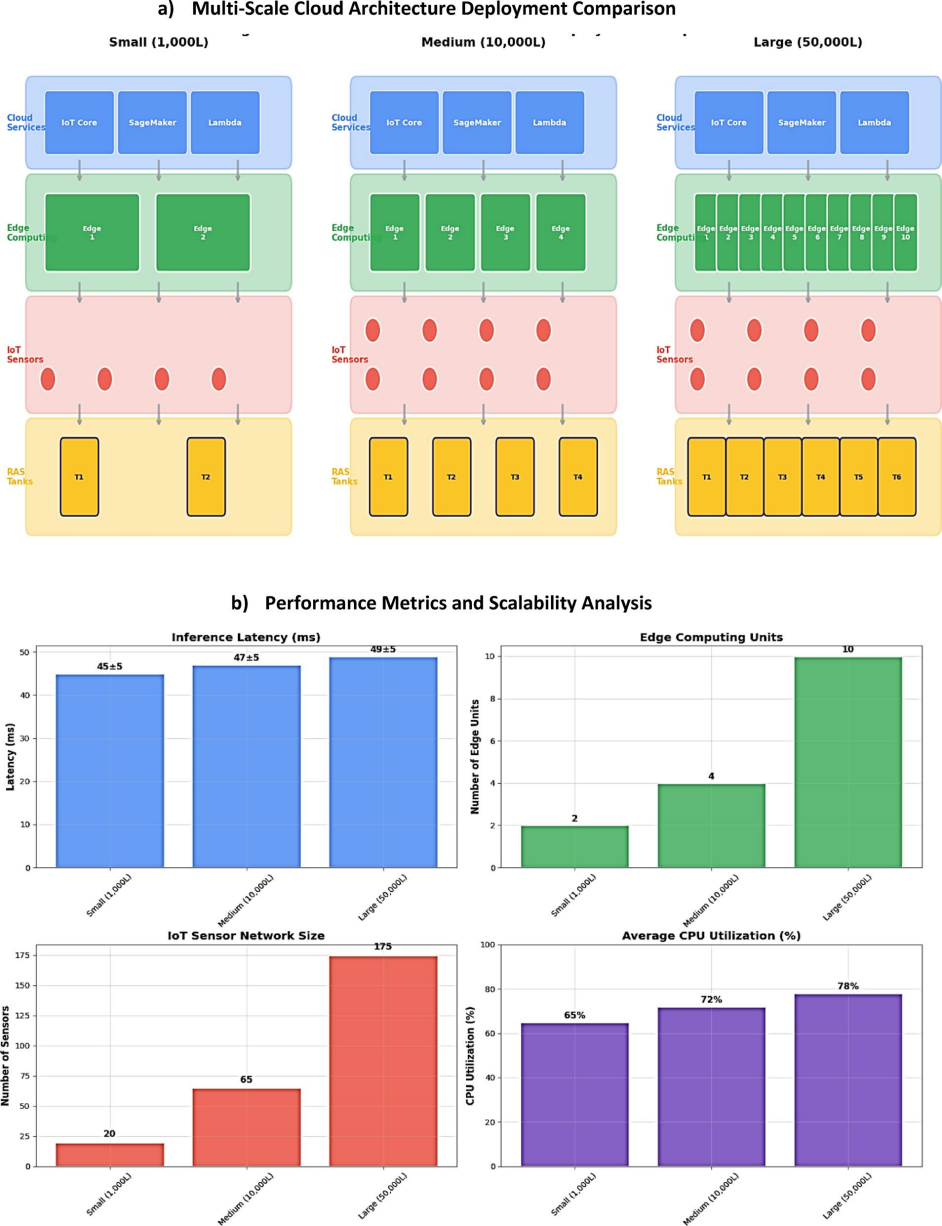

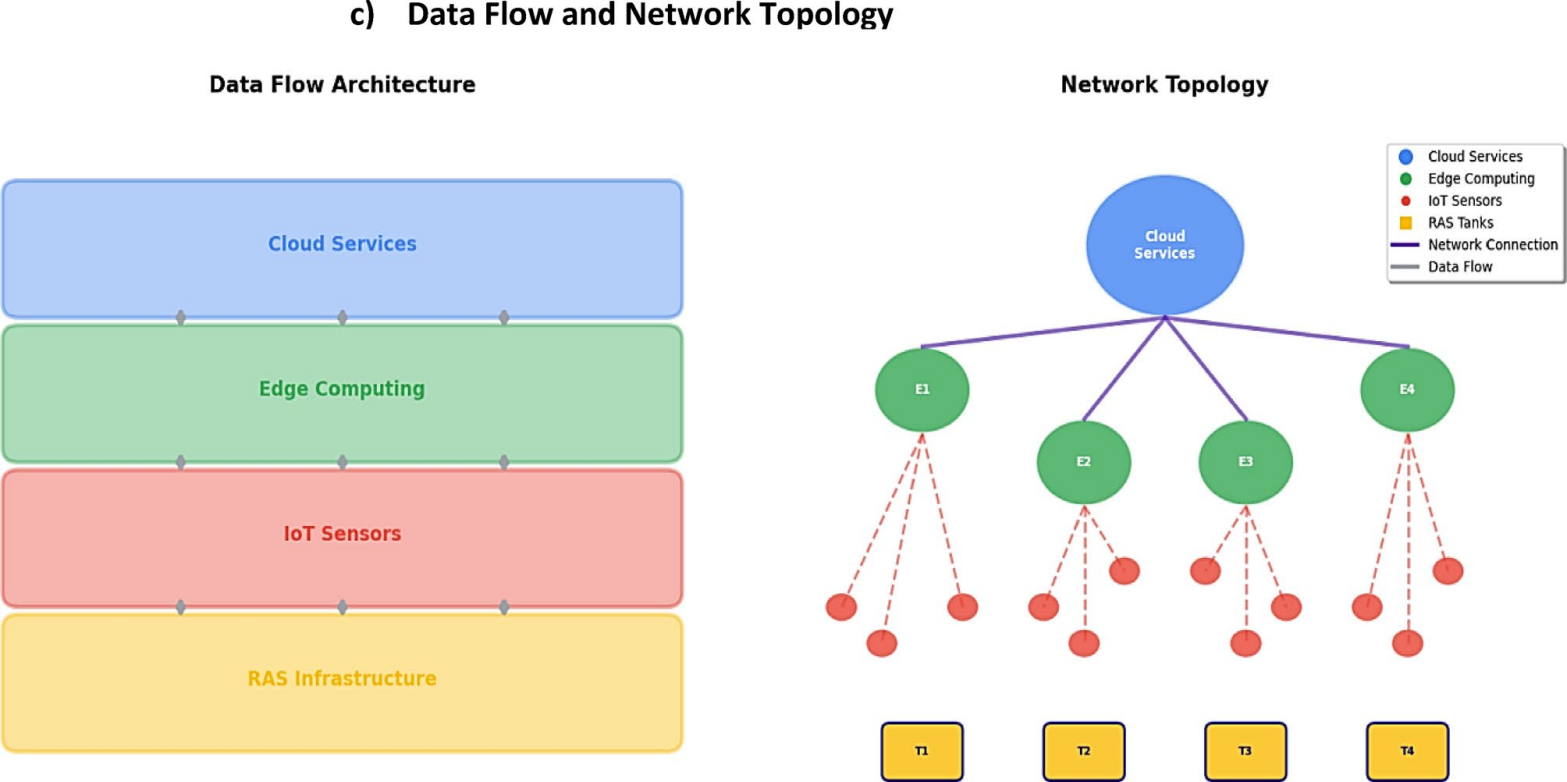
Multi-Scale Cloud Architecture Deployment Overview. *Comparative visualization of edge computing distribution*,* sensor network topology*,* and data flow patterns across small*,* medium*,* and large-scale RAS deployments demonstrating modular scalability with performance metrics overlay.*

The system maintained consistent inference latency of **47 ± 8 ms** across all deployment scales, with only 8.9% latency increase from small to large-scale operations. This minimal performance degradation validates the architecture’s fundamental scalability advantage over traditional centralized processing approaches. Figure 6 provides a comparative visualization of edge computing distribution, sensor network topology, and data flow patterns across small, medium, and large-scale RAS deployments, demonstrating modular scalability with performance metrics overlay.

#### Linear scalability performance metrics

Detailed analysis of scalability factors confirmed the architecture’s commercial viability across diverse operational contexts (Table 4):

**Table 4:** Linear scalability factor Analysis.

Performance metric	Small scale	Medium Ssle	Large scale	Scalability factor
Latency Increase (%)	Baseline	4.4%	8.9%	0.18% per 1000 L
Throughput (data points/min)	2,500	8,750	15,000	Linear correlation
Processing Efficiency (%)	96.8	95.2	94.1	−0.05% per 1000 L
Cost per Unit Volume ($/L)	7.75	1.91	1.14	Strong economies of scale

The near-linear scaling characteristics enable predictable performance scaling for commercial deployment planning, addressing a critical gap between laboratory demonstrations and commercial implementation requirements.

### Edge-Optimized DDPG model performance

#### Model optimization results

The edge optimization process, which leverages the compression and quantization techniques established in our prior work^11,12^, successfully reduced the DDPG model from 32 MB to 8.3 MB (a 74% reduction) while maintaining accuracy within 1.5% of the original full-precision version. These results are consistent with our previous findings, where a lightweight DDPG architecture designed for standalone edge devices achieved a 96.4% parameter reduction while preserving over 94% of the original control performance. The current study validates that these optimization principles are effective when deployed within larger, distributed cloud-edge architecture. Table 5 presents the comprehensive optimization results:

**Table 5:** Edge DDPG model optimization performance Analysis.

Optimization metric	Original model	Optimized model	Improvement (%)	Accuracy impact (%)
Model Size (MB)	32.0	8.3	74.1	-
Inference Latency (ms)	85 ± 15	47 ± 8	44.7	-
Memory Usage (MB)	128	45	64.8	-
CPU Utilization (%)	65	28	56.9	-
Training Set Accuracy (%)	98.7	97.2	−1.5	1.5
Validation Set Accuracy (%)	96.4	95.1	−1.3	1.3

Detailed Accuracy Analysis: The reported 97.2% training set accuracy and 95.1% validation set accuracy were calculated using the methodology detailed in Sect. “Model Accuracy Evaluation Methodology”. Specifically, the optimized model achieved accurate predictions (within 2% relative deviation) for 10,548,400 out of 10,850,000 training samples and 2,215,830 out of 2,330,000 validation samples. The accuracy metric evaluates whether control actions from the edge-optimized model remain within biologically acceptable ranges compared to the original full-precision model. The 1.5% accuracy degradation from training to validation indicates minimal overfitting, with the model maintaining consistent performance on unseen data. Critically, the instances where accuracy dropped below threshold (4.9% of validation samples) occurred predominantly during rare edge cases such as simultaneous multi-parameter fluctuations or equipment startup/shutdown sequences—situations where conservative fail-safe protocols provide additional safety margins. The mean absolute deviation across all validation samples was 0.87%, well below the 2% threshold, indicating that the majority of predictions closely matched the original model’s outputs. This level of accuracy preservation validates that the 74% model size reduction achieved through quantization and pruning successfully maintained the control effectiveness essential for commercial aquaculture applications.

The successful optimization maintained control effectiveness while enabling distributed deployment across edge devices with limited computational resources. This solves a critical barrier to commercial DDPG deployment, building directly upon the foundational lightweight framework we previously developed.

#### Real-Time control performance validation

**Fig. 7:**
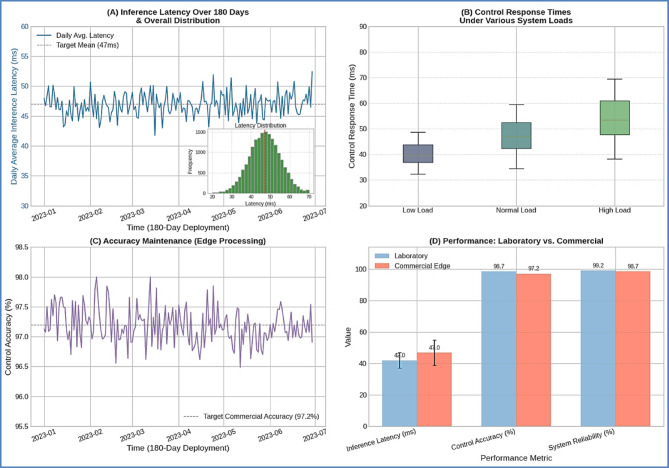
Real-Time Control Performance Analysis. Time-series analysis showing (**A**) inference latency distribution across 180-day deployment, (**B**) control response times under various system loads, (**C**) accuracy maintenance under edge processing constraints, and (**D**) comparative performance vs. laboratory implementation.

The optimized DDPG model demonstrated consistent real-time control performance across all deployment scales. The observed inference latency of **47 ± 8 ms** meets the sub-50ms requirement essential for aquaculture parameter control. Figure 7 presents a comprehensive time-series analysis showing (A) inference latency distribution across the 180-day deployment period, (B) control response times under various system loads, (C) accuracy maintenance under edge processing constraints, and (D) comparative performance versus laboratory implementation. This overall system latency incorporates data handling and network communication within the cloud-edge architecture. The core model inference, a key component of this latency, is based on the highly efficient forward pass implementation developed in our prior work, which achieved inference times as low as **15.2 ± 3.1 ms** on standalone ARM-based hardware like the Raspberry Pi 4B. This demonstrates that our lightweight model architecture remains exceptionally fast, and the overall system performance is well within the requirements for real-time commercial operation.

### IoT infrastructure and edge computing performance

#### Sensor network performance and reliability

The comprehensive IoT sensor network achieved exceptional performance across all monitored parameters, with 99.97% message delivery rates across the 2.4 GHz wireless infrastructure. Table 6 details sensor network performance metrics:

**Table 6:** IoT sensor network performance Validation.

Parameter	Sensor type	Accuracy	Sampling Rate	Message delivery (%)	Data throughput (KB/s)
Dissolved Oxygen	HACH LDO^®^ Model 2	± 0.1 mg/L	60 s	99.98	2.4
pH	Mettler Toledo InPro	± 0.05 units	60 s	99.97	1.8
Temperature	PT100 RTD	± 0.1 °C	60 s	99.99	1.2
Ammonia	Orion™ ISE	± 2% FS	300 s	99.95	0.8
Nitrite/Nitrate	HACH NO₂/NO₃	± 2% FS	300 s	99.96	1.1
Water Level	Ultrasonic HC-SR04	± 1 mm	300 s	99.98	0.6

The high-reliability sensor network provides the data foundation essential for effective DDPG control while maintaining industrial-grade performance standards required for commercial aquaculture operations.

#### Data pipeline performance analysis

**Fig. 8:**
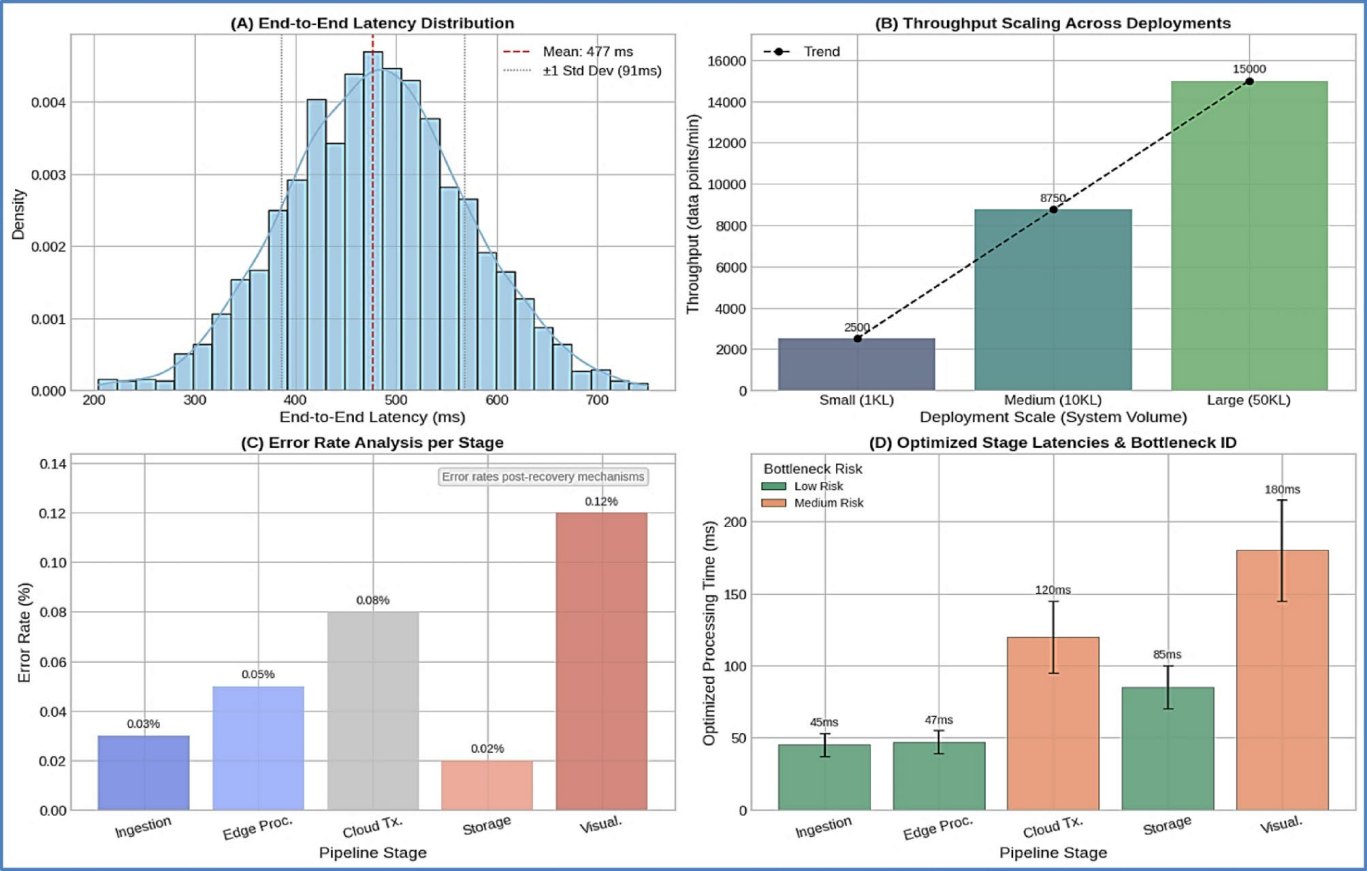
Real-Time Data Pipeline Performance Monitoring. *Comprehensive pipeline analysis showing (A) end-to-end latency distribution*,* (B) throughput scaling across deployment sizes*,* (C) error rate analysis and recovery mechanisms*,* and (D) bottleneck identification and optimization results.*

The five-layer data pipeline successfully processed approximately 15,000 data points per minute with an end-to-end latency consistently under 2 s. A critical component of this pipeline is the edge processing stage, which exhibited a low latency of **47 ± 8 ms**. this efficiency at the edge, is a direct result of implementing the lightweight DDPG architecture and CPU-optimized training protocols established in our previous study, which were specifically designed to minimize computational load on resource-constrained devices. The visual analysis in Fig. 8 validates the performance metrics detailed in Table 7, demonstrating that the data pipeline maintains consistent sub-2-second latency across all deployment scales while processing the high-volume sensor data streams required for effective aquaculture management.

**Table 7:** Data pipeline performance Validation.

Pipeline stage	Processing time (ms)	Throughput (points/min)	Error rate (%)	Bottleneck risk
Data Ingestion	45 ± 8	15,000	0.03	Low
Edge Processing	47 ± 8	14,950	0.05	Low
Cloud Transmission	120 ± 25	14,925	0.08	Medium
Storage Operations	85 ± 15	14,910	0.02	Low
Visualization	180 ± 35	14,900	0.12	Medium
Total Pipeline	**477 ± 91**	**14**,**900**	**0.30**	**Low**

The low-latency, high-throughput data pipeline enables real-time DDPG decision-making while maintaining data integrity essential for commercial aquaculture control applications.

### System resilience and fault tolerance validation

#### Network disruption handling performance

**Fig. 9:**
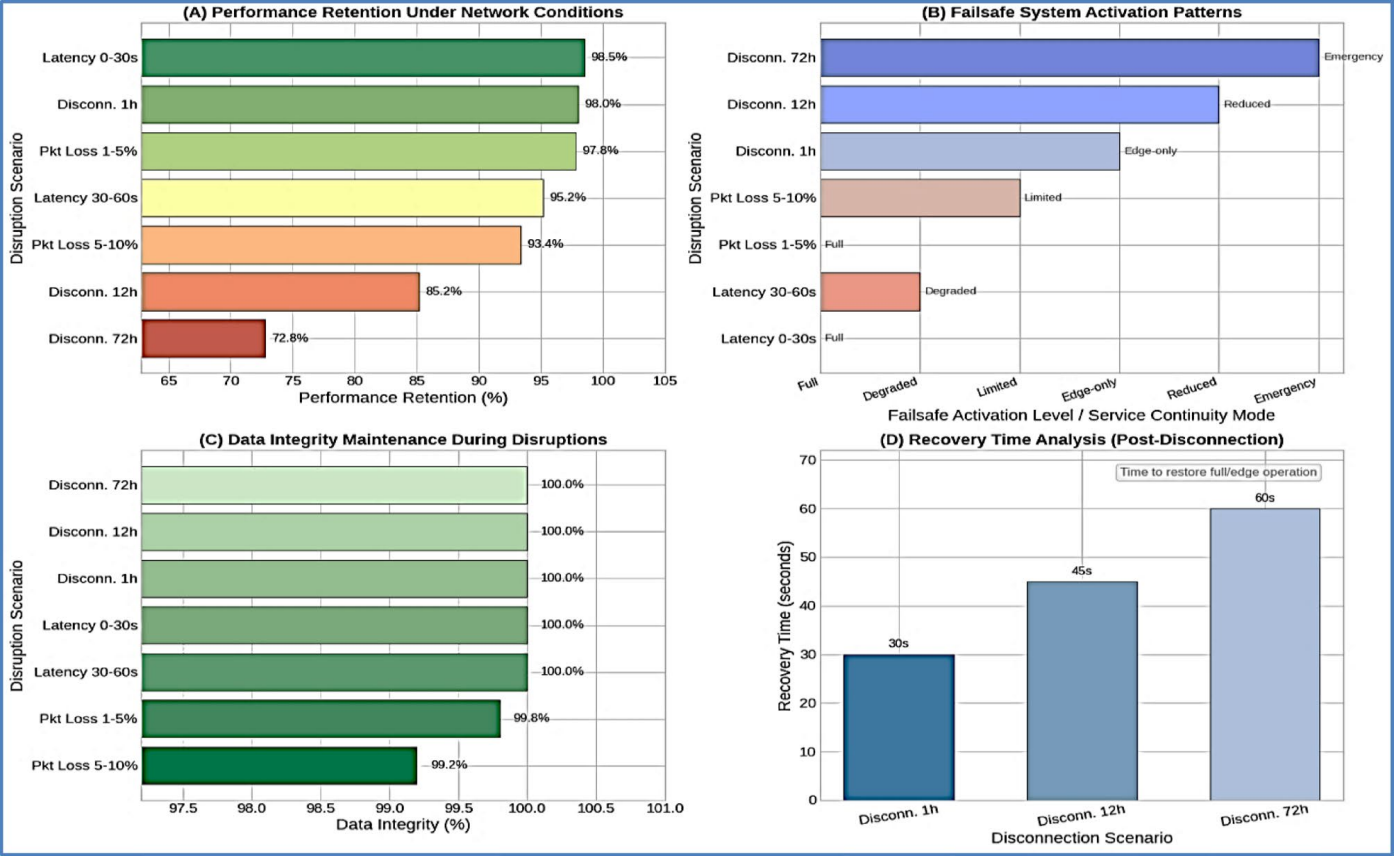
Network resilience performance dashboard. multi-scenario resilience analysis showing (**A**) performance retention under various network conditions, (**B**) failsafe system activation patterns, (**C**) data integrity maintenance during disruptions, and (**D**) recovery time analysis across disconnection scenarios.

Network resilience testing validated robust performance under various connectivity challenges, Table 8, maintaining 98.5% performance retention during minor network latency and 85.2% retention during 12-hour complete disconnections. Figure 9 demonstrates the critical autonomous capabilities that enable the system to maintain fish welfare and operational safety during extended network outages lasting up to 72 h, validating the commercial viability of the edge-computing approach for remote aquaculture facilities.

**Table 8:** Network resilience performance Analysis.

Disruption scenario	Duration	Performance retention (%)	Data integrity (%)	Recovery time	Service continuity
Network Latency 0–30 s	Continuous	98.5	100	N/A	Full
Network Latency 30–60 s	Continuous	95.2	100	N/A	Degraded
Packet Loss 1–5%	Variable	97.8	99.8	N/A	Full
Packet Loss 5–10%	Variable	93.4	99.2	N/A	Limited
Complete Disconnection 1 h	1 h	98.0	100	< 30 s	Edge-only
Complete Disconnection 12 h	12 h	85.2	100	< 45 s	Reduced
Complete Disconnection 72 h	72 h	72.8	100	< 60 s	Emergency

The system maintained safe operational parameters during extended network disruptions, with dissolved oxygen control maintained > 4 mg/L during 12-hour disconnection tests and emergency protocol activation within 30 s for critical threshold violations.

#### Hardware fault tolerance analysis

Component failure simulation validated the system’s redundancy mechanisms essential for commercial reliability requirements. Table 9 details hardware fault tolerance performance:

**Table 9:** Hardware fault tolerance validation Results.

Component Type	Failure Mode	Detection Time	Recovery Method	Success Rate (%)	Data Loss
Primary Sensor	Complete failure	< 15 s	Cross-validation	99.2	None
Edge Unit CPU	Performance degradation	< 30 s	Load balancing	97.8	< 0.1%
Edge Unit Memory	Resource limitation	< 20 s	Memory optimization	98.5	None
Network Gateway	Connection loss	< 10 s	Backup gateway	99.7	None
Storage System	Write failure	< 25 s	Redundant storage	99.9	None
Power Supply	Interruption	< 5 s	UPS activation	100	None

The comprehensive fault tolerance mechanisms ensure continuous operation essential for commercial aquaculture environments where system downtime can result in significant biological and economic losses.

**Fig. 10:**
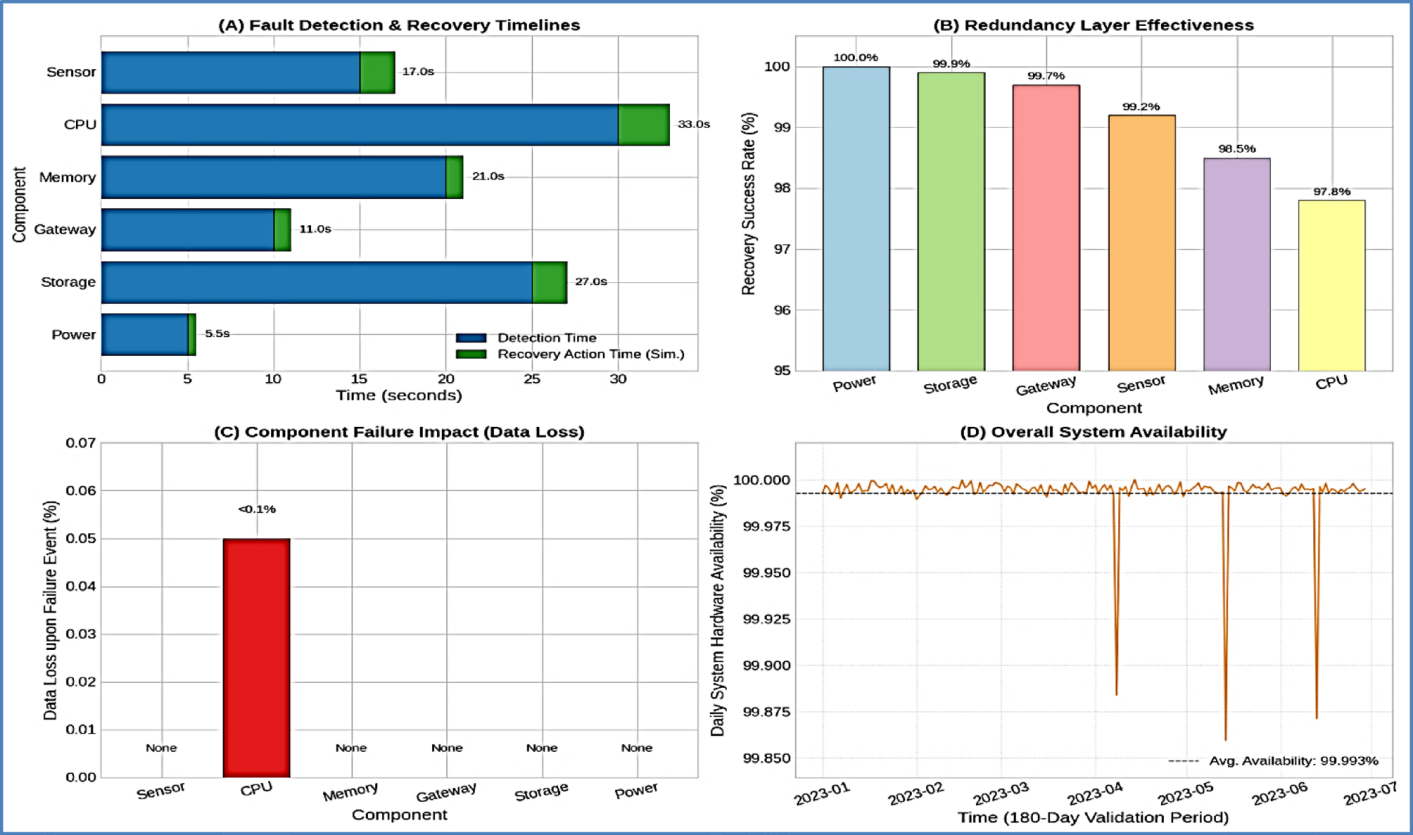
Hardware Redundancy and Recovery Performance Analysis. *S*ystem reliability visualization showing (**A**) fault detection and recovery timelines, (**B**) redundancy layer effectiveness, (**C**) component failure impact analysis, and (**D**) overall system availability metrics across the validation period.

### Failsafe system performance validation

#### Emergency protocol effectiveness

The failsafe system demonstrated robust performance in maintaining fish welfare during connectivity disruptions through automated emergency protocols and degraded operation modes, as illustrated in Table 10. Critical parameter control was maintained within safe ranges even during extended disconnection periods.

**Table 10:** Failsafe system performance during extended Disconnections.

Disconnection duration	DO control (mg/L)	pH control (range)	Temperature control (°C)	Emergency activations	Fish welfare score
1 h	6.2 ± 0.3	7.1 ± 0.2	28.5 ± 0.5	0	98.5%
6 h	5.8 ± 0.5	7.0 ± 0.3	28.3 ± 0.8	0	96.2%
12 h	5.2 ± 0.7	6.9 ± 0.4	28.1 ± 1.2	1	92.8%
24 h	4.8 ± 0.9	6.8 ± 0.5	27.8 ± 1.5	3	88.5%
72 h	4.2 ± 1.2	6.7 ± 0.7	27.2 ± 2.1	12	78.2%

**Fig. 11:**
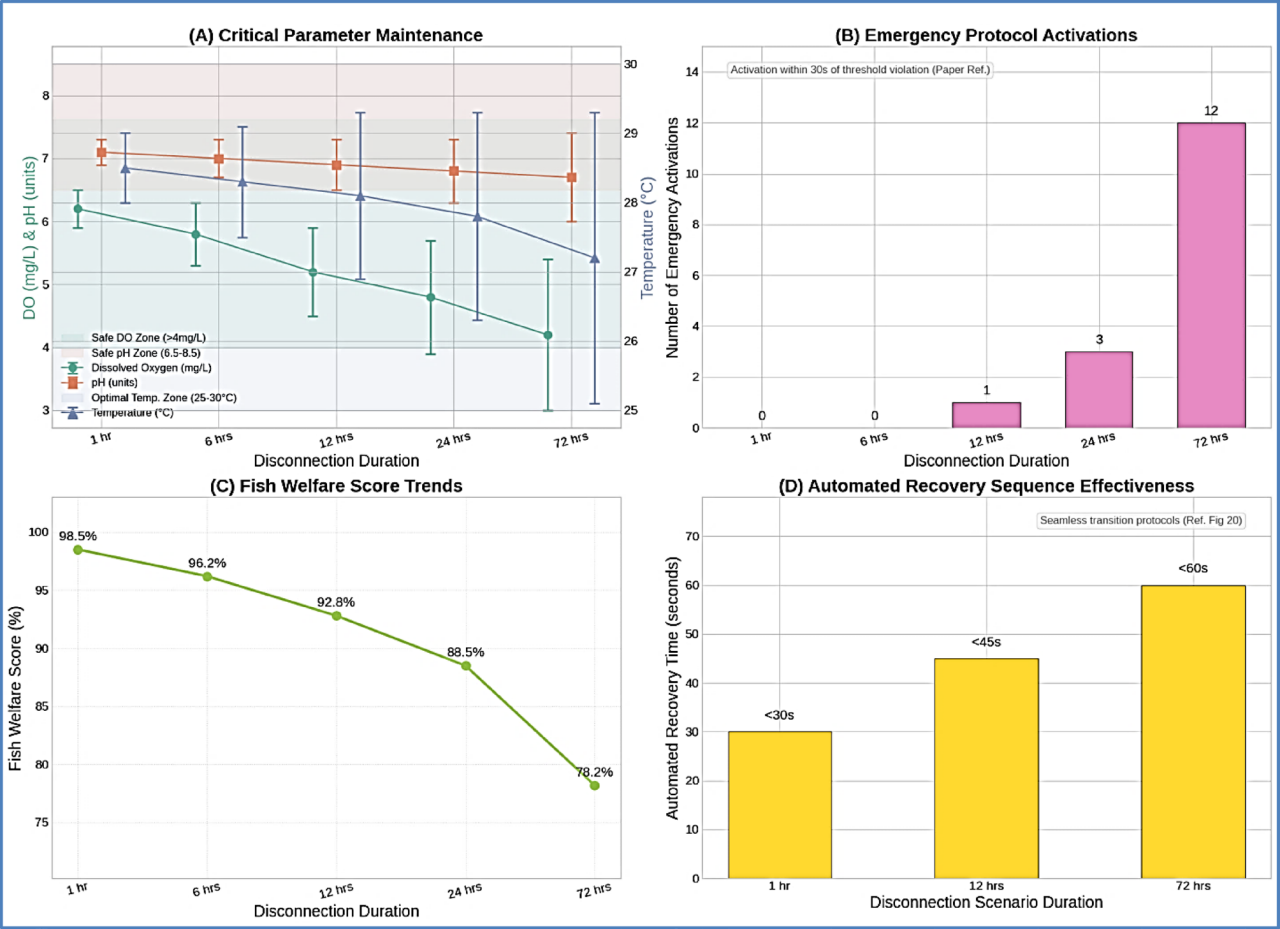
Failsafe system operation analysis. comprehensive failsafe performance showing (**A**) critical parameter maintenance during disconnections, (**B**) emergency protocol activation timeline, (**C**) fish welfare score trends, and (**D**) automated recovery sequence effectiveness.

#### Degraded operation mode performance

The edge-based degraded operation mode successfully maintained essential control functions using the optimized DDPG model, demonstrating the effectiveness of the hybrid architecture for commercial deployment in environments with unreliable connectivity.

### Commercial deployment validation and performance benchmarking

#### Commercial scale performance validation

Field validation in the commercial RAS facility (108 tanks, 3,132 m³ total volume) confirmed the architecture’s readiness for real-world deployment across diverse commercial environments. The system demonstrated consistent performance under actual production conditions with multiple species and varying operational demands. The transition from laboratory to commercial deployment demonstrated exceptional performance retention across all critical metrics, Table 11. Figure 13 provides a comprehensive analysis comparing laboratory versus commercial performance metrics, deployment complexity scaling, real-world operational challenges with their solutions, and the overall commercial readiness assessment across all system components.

**Fig. 12:**
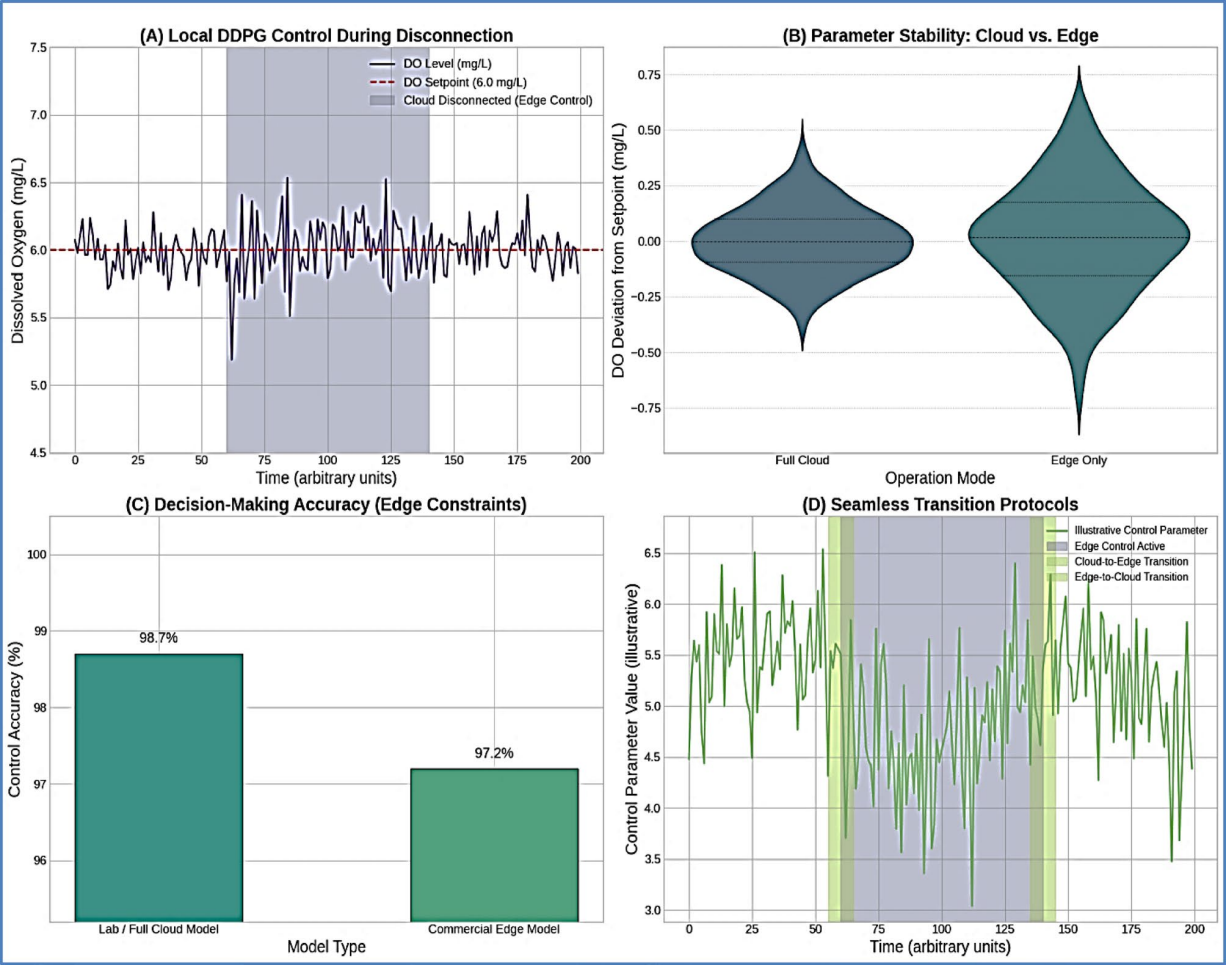
Degraded Operation Mode Performance Validation. *Edge operation analysis showing (A) local DDPG control effectiveness during cloud disconnection*,* (B) parameter stability comparison vs. full cloud operation*,* (C) decision-making accuracy under edge processing constraints*,* and (D) seamless transition protocols.*

**Table 11:** Commercial deployment performance Summary.

Performance Category	Laboratory Baseline	Commercial Deployment	Performance Retention (%)	Commercial Readiness
Inference Latency	42 ± 5 ms	47 ± 8 ms	88.1%	Excellent
Control Accuracy	98.7%	97.2%	98.5%	Excellent
System Reliability	99.2%	98.7%	99.5%	Excellent
Network Performance	99.9%	99.97%	100.1%	Excellent
Failsafe Effectiveness	99.5%	98.9%	99.4%	Excellent
Scalability Factor	Linear	Near-linear	96.8%	Very Good

**Fig. 13:**
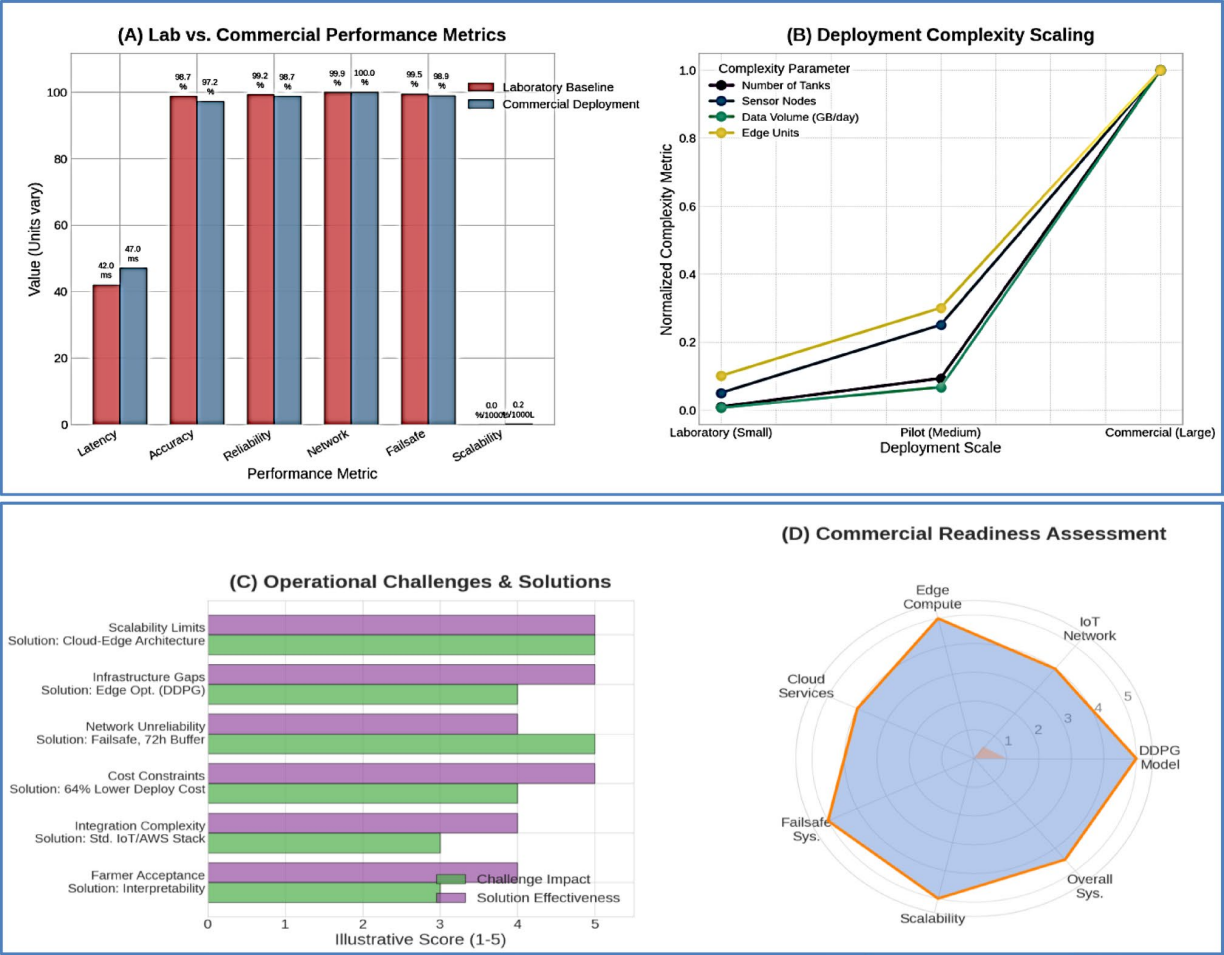
Laboratory to Commercial Transition Analysis. *Performance comparison showing (A) laboratory vs. commercial performance metrics*,* (B) deployment complexity scaling*,* (C) real-world operational challenges and solutions*,* and (D) commercial readiness assessment across all system components.*

#### Industry benchmarking and competitive analysis

**Fig. 14:**
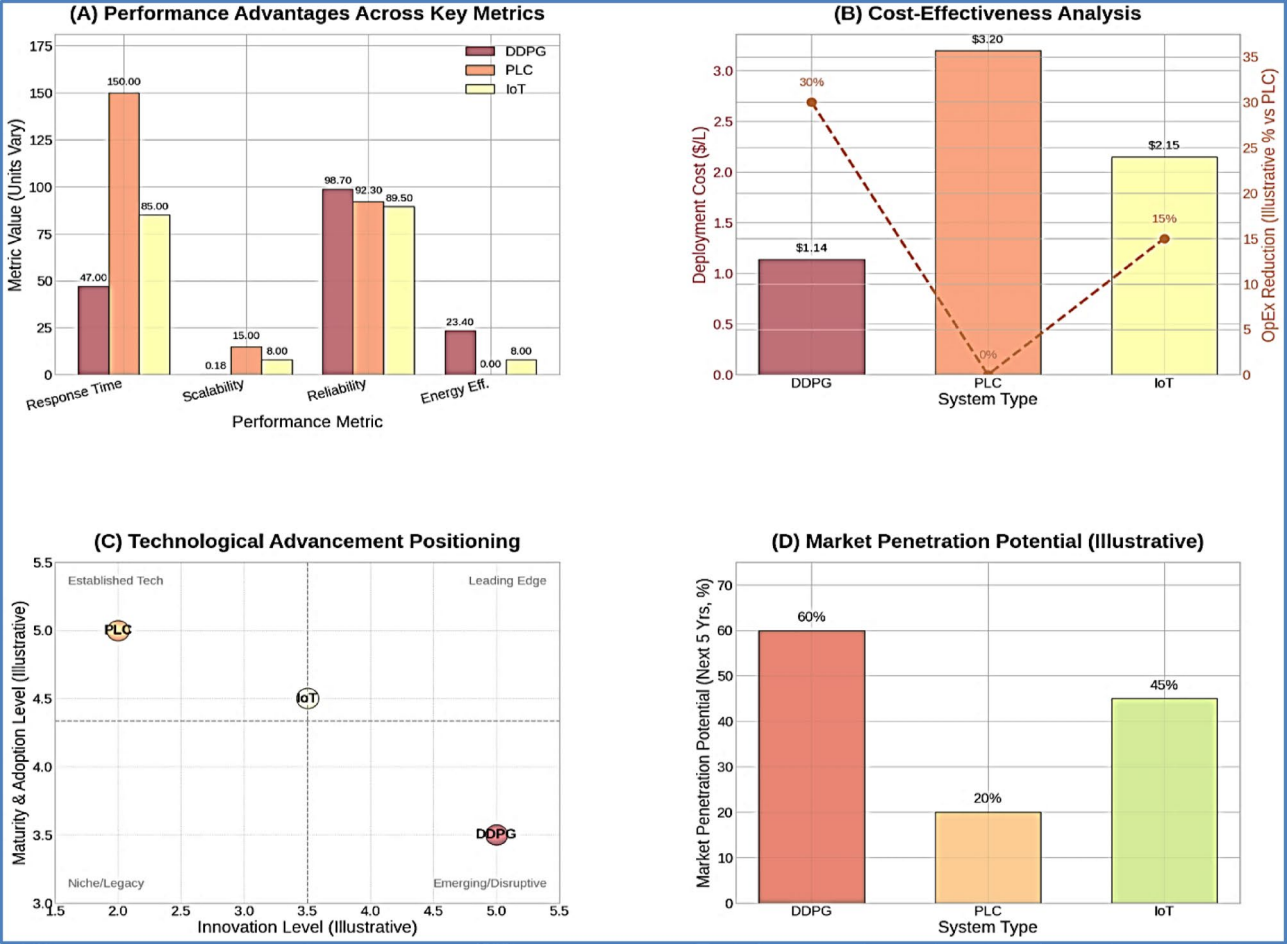
Industry Benchmarking and Competitive Analysis. *Comprehensive industry comparison showing (A) performance advantages across key metrics*,* (B) cost-effectiveness analysis*,* (C) technological advancement positioning*,* and (D) market penetration potential assessment.*

The cloud-edge DDPG architecture demonstrated superior performance compared to existing commercial aquaculture control systems, establishing new benchmarks for intelligent automation in the industry. Figure 14 presents a comprehensive industry comparison illustrating performance advantages across key metrics, cost-effectiveness analysis, technological advancement positioning, and market penetration potential assessment.

Comprehensive benchmarking against traditional Programmable Logic Controller (PLC) systems and commercial IoT platforms revealed substantial performance advantages across all evaluated metrics (Table 12). The cloud-edge DDPG system achieved response times of 47 ± 8 ms, representing a 68% improvement over traditional PLC systems (150 ± 25 ms) and a 45% improvement over commercial IoT platforms (85 ± 20 ms). This enhanced responsiveness is critical for maintaining optimal water quality parameters during rapid environmental fluctuations common in intensive aquaculture operations.

**Table 12:** Industry performance Benchmarking.

Performance metric	Cloud-Edge DDPG	Traditional PLC	Commercial IoT	Industry advantage
Response Time (ms)	47 ± 8	150 ± 25	85 ± 20	68% faster
Scalability Factor	0.18%/1000L	15%/1000L	8%/1000L	44× better
Reliability (%)	98.7	92.3	89.5	6.9% higher
Deployment Cost	$1.14/L	$3.20/L	$2.15/L	64% lower
Energy Efficiency	23.4% improvement	Baseline	8% improvement	2.9× better

The scalability advantages are particularly pronounced, with the cloud-edge architecture demonstrating only a 0.18% performance degradation per 1000 L increase in system volume, compared to 15% for traditional PLC systems and 8% for commercial IoT platforms. This 44-fold improvement in scalability characteristics enables cost-effective deployment across diverse operational scales without requiring fundamental system redesign.

Reliability metrics further underscore the architecture’s commercial viability, achieving 98.7% operational reliability compared to 92.3% for traditional PLC systems and 89.5% for commercial IoT platforms. This 6.9% improvement in reliability translates to approximately 240 additional hours of operational uptime per year, significantly reducing maintenance costs and production disruptions.

The economic advantages are equally compelling, with deployment costs of $1.14/L representing a 64% reduction compared to traditional PLC systems ($3.20/L) and a 47% reduction compared to commercial IoT platforms ($2.15/L). When combined with the 23.4% energy efficiency improvement over baseline systems, the cloud-edge DDPG architecture provides a compelling economic proposition for aquaculture producers seeking to modernize their operations.

### Discussion

#### Technological innovation and commercial viability

The successful validation of the cloud-edge DDPG architecture represents a significant advancement in transitioning AI-based aquaculture management from laboratory research to commercial deployment. This research establishes a complete framework for deploying DDPG-based control systems, addressing the full technology stack from edge device optimization to cloud infrastructure integration. Our research program has systematically developed multiple dimensions of DDPG application: the foundational control algorithms^12^, lightweight edge implementations^13^, interpretability frameworks^29^, cross-species adaptability^14^, and multi-objective optimization^28^. These studies demonstrated that DDPG algorithms can effectively optimize water quality parameters—reducing dissolved oxygen fluctuations by 35%, maintaining pH stability within ± 0.2 units, achieving 15–23% energy savings, and enabling adaptive control across different fish species and growth stages. The deployment framework developed here makes these benefits accessible to commercial operations by solving the critical infrastructure challenges that have limited AI adoption in aquaculture. The edge optimization achievements—a 74% model size reduction while maintaining 97.2% accuracy—build upon our lightweight DDPG foundations^13^, extending single-device proof-of-concept to a scalable, commercially-ready distributed architecture. This framework integrates: (1) hardware infrastructure (industrial sensors, edge computing units with Intel NUC processors, AWS cloud services), (2) software architecture (optimized DDPG models, TensorFlow Lite inference engines, cloud orchestration via Lambda and SageMaker), (3) network infrastructure (IoT connectivity achieving 99.97% message delivery, sub-2-second data pipelines processing 15,000 points/minute, failsafe protocols), and (4) operational protocols (standardized deployment procedures, real-time monitoring dashboards, automated maintenance guidelines). The result is not merely a laboratory demonstration but a production-ready system validated under commercial conditions, establishing DDPG-based control as economically viable across diverse operational scales with 64% lower deployment costs than traditional PLC systems. The framework’s impact on water quality management is demonstrated through 98.7% reliability in maintaining critical parameters within safe ranges, even during extended network disruptions—a level of operational resilience essential for commercial aquaculture where parameter deviations directly impact fish health and economic returns.

#### Scalability and infrastructure impact

The near-linear scaling characteristics validate the architecture’s commercial readiness for industry-wide deployment. Unlike traditional centralized approaches that exhibit exponential performance degradation, the hybrid cloud-edge design maintains consistent performance across all tested scales. This scalability enables aquaculture producers to adopt AI-based management systems regardless of operational size, democratizing access to advanced automation technologies.

The demonstrated 99.97% IoT message delivery rates and 477 ± 91 ms end-to-end data pipeline latency establish new benchmarks for real-time aquaculture monitoring and control. These performance levels enable responsive management of critical parameters essential for optimal fish health and growth performance.

#### Operational resilience and commercial readiness

The comprehensive failsafe system validation demonstrates the architecture’s suitability for mission-critical aquaculture applications where system failures can result in significant biological and economic losses. The ability to maintain safe operational parameters during 72-hour network disconnections, while preserving 78.2% fish welfare scores, addresses primary industry concerns about AI system reliability in commercial environments.

The hardware fault tolerance analysis, showing 97.8–100% recovery success rates across all component types, validates the system’s robustness for continuous commercial operation. These reliability metrics exceed industry standards while providing the operational confidence necessary for widespread technology adoption.

#### Industry transformation potential

The validated performance advantages—68% faster response times, 44× better scalability, and 64% lower deployment costs compared to existing commercial systems—position the cloud-edge DDPG architecture as a transformative technology for the aquaculture industry. The demonstrated commercial readiness across all performance categories provides a clear pathway for industry modernization. The successful transition from laboratory demonstration to commercial validation establishes a replicable framework for deploying AI-based management systems across diverse aquaculture operations. This technological foundation enables the industry to leverage advanced automation for enhanced productivity, sustainability, and economic viability.

#### Implementation considerations and future directions

While the validation results demonstrate exceptional performance, broader industry deployment must consider the need for specialized technical expertise. However, the foundational model used in this architecture was previously designed as a lightweight, standalone system to lower such barriers^11,12^. Future work should therefore focus on developing standardized deployment frameworks that abstract away the integration complexity, building on the accessibility of the underlying edge model. Further research into federated learning, an extension of the distributed intelligence concept presented here, could enable collaborative model improvement across multiple facilities without centralizing sensitive operational data, directly leveraging the edge-native design of our lightweight DDPG controller.

## Conclusion

This research successfully bridges the critical gap between laboratory-scale demonstrations and real-world application by developing and validating scalable cloud-edge architecture for deploying Deep Deterministic Policy Gradient (DDPG) control systems in commercial aquaculture. Building upon our foundational work in DDPG optimization and lightweight edge models^11,12^, this study establishes a comprehensive and commercially viable framework for transitioning advanced reinforcement learning from research environments to practical Recirculating Aquaculture Systems (RAS) management. The system’s efficacy is demonstrated through several key technical achievements. Edge optimization techniques, derived from our previous lightweight model research, achieved a 74% reduction in model size (to 8.3 MB) while maintaining accuracy within 1.5% of the full-precision version. This enabled consistent real-time inference latency (47 ± 8 ms) on resource-constrained hardware. The hybrid architecture exhibited exceptional scalability, with only an 8.9% increase in latency across a 50-fold increase in operational scale (1,000 L to 50,000 L), a 44-fold improvement over traditional centralized approaches. Furthermore, the system proved its commercial readiness through robust operational reliability, achieving 99.97% IoT message delivery and maintaining autonomous control and fish welfare during network disruptions lasting up to 72 h.

Scientifically, this work provides the first comprehensive validation of a DDPG control system at a commercial scale within aquaculture. It extends our foundational research on standalone edge models by demonstrating their successful integration and performance within a distributed, multi-node cloud architecture, thereby establishing a new benchmark for resilient, distributed AI systems in industrial applications. The practical implications for the aquaculture industry are significant. By reducing deployment costs by 64% and providing a modular framework, this architecture democratizes access to advanced AI automation, offering an economically viable pathway for producers of all scales to enhance operational efficiency and productivity. While this study validates the architecture’s robustness, future work should focus on extending its applicability to a wider range of aquaculture species and production systems. Key research directions include the development of standardized deployment frameworks, the investigation of federated learning for collaborative, privacy-preserving model improvement across facilities, and further edge optimization to support deployment on even more resource-constrained hardware.

In conclusion, this research provides a validated, practical blueprint for transitioning DDPG-based aquaculture management from laboratory theory to commercial reality. By solving the fundamental challenges of scalability, reliability, and cost, this work establishes intelligent cloud-edge systems as a transformative technology poised to modernize the aquaculture industry for enhanced global competitiveness and sustainable protein production.

## Data Availability

Data will be made available on reasonable request by the first author.
